# Case Report: A Chinese Family of Woodhouse-Sakati Syndrome With Diabetes Mellitus, With a Novel Biallelic Deletion Mutation of the DCAF17 Gene

**DOI:** 10.3389/fendo.2021.770871

**Published:** 2021-12-23

**Authors:** Min Zhou, Ningjie Shi, Juan Zheng, Yang Chen, Siqi Wang, Kangli Xiao, Zhenhai Cui, Kangli Qiu, Feng Zhu, Huiqing Li

**Affiliations:** ^1^ Department of Pulmonary and Critical Care Medicine, Tongji Hospital, Tongji Medical College, Huazhong University of Science and Technology, Wuhan, China; ^2^ Key Laboratory of Respiratory Diseases, National Ministry of Health of the People’s Republic of China and National Clinical Research Center for Respiratory Disease, Wuhan, China; ^3^ Department of Endocrinology, Union Hospital, Tongji Medical College, Huazhong University of Science and Technology, Wuhan, China; ^4^ Hubei Provincial Clinical Research Center for Diabetes and Metabolic Disorders , Wuhan, China; ^5^ Clinic Center of Human Gene Research, Union Hospital, Tongji Medical College, Huazhong University of Science and Technology, Wuhan, China; ^6^ Department of Cardiology, Union Hospital, Tongji Medical College, Huazhong University of Science and Technology, Wuhan, China

**Keywords:** Woodhouse–Sakati syndrome, diabetes, intellectual disability, alopecia, hypogonadism

## Abstract

Woodhouse–Sakati syndrome (WSS) (OMIM#241080) is a rare multi-system autosomal recessive disease with homozygous mutation of the DCAF17 gene. The main features of WSS include diabetes, hypogonadism, alopecia, deafness, intellectual disability and progressive extrapyramidal syndrome. We identified a WSS family with a novel DCAF17 gene mutation type in China. Two unconsanguineous siblings from the Chinese Han family exhibiting signs and symptoms of Woodhouse-Sakati syndrome were presented for evaluation. Whole-exome sequencing revealed a homozygous deletion NM_025000.4:c.1488_1489delAG in the DCAF17 gene, which resulted in a frameshift mutation that led to stop codon formation. We found that the two patients exhibited low insulin and C-peptide release after glucose stimulation by insulin and C-peptide release tests. These findings indicate that the DCAF17 gene mutation may cause pancreatic β cell functional impairment and contribute to the development of diabetes.

## Introduction

WSS, which is a rare autosomal recessive genetic disease, was first reported in 1983 (1). Previous, reports revealed the presence of hypogonadism (100%), diabetes mellitus (66%), intellectual disability (58%), sensorineural hearing loss (62%), and extrapyramidal movements (65%) (2). To date, 88 cases from more than 40 families have been reported (2). Most of these families originate from the Middle East (3, 4), particularly Saudi Arabia. Some cases have also been recognized in other areas such as Europe, Turkey, India, Pakistan, Portugal, France, and Japan. The disease is caused by mutations in the DCAF17 gene (also known as C2orf37), which is located on chromosome 2q22.3-q35 (5). The DCAF17 gene contains at least 14 exons and encodes a nuclear transmembrane protein whose specific function is unclear (6). Eighteen types of pathogenic mutations have been identified, including three nonsense, five intronic, nine frameshift and one start loss mutation, all of which result in truncated nonfunctional protein products. Based on the hypothesis of Alazami et al. (2008), the mutation of DCAF17 impairs the function of the nucleolus, which causes disruptions of normal cell routines such as cell cycle regulation, cellular senescence, and apoptosis, which may underlie the pathogenesis of WSS (5).

We identified a WSS family in China and found a novel DCAF17 deletion mutation *via* genetic testing. Two patients in this family were affected with clinical features of diabetes, alopecia, intellectual disability, hypogonadism, anemia and thrombocytopenia.

## Case Description

### Case 1

**Figure 1:**
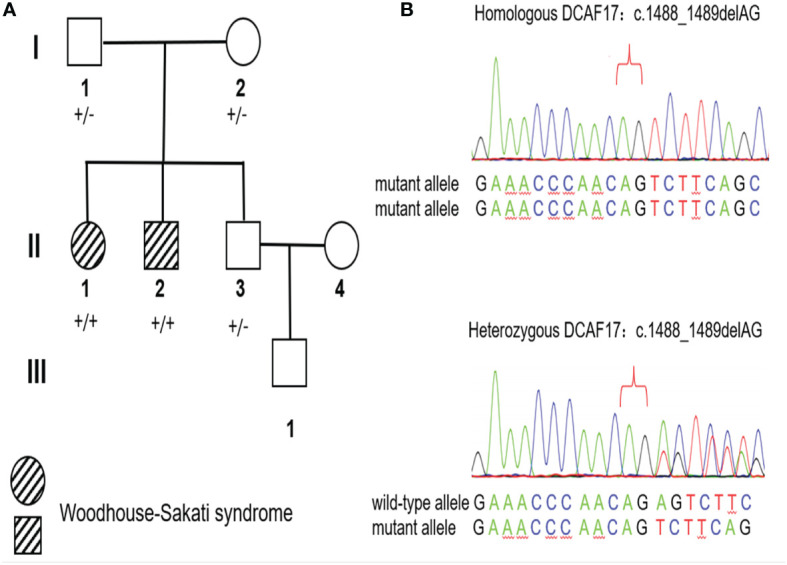
Pedigree and Sanger sequencing chromatograms of the identified disease-causing variants. **(A)** Pedigree of the WSS family. Marks ‘+/+’ and ‘+/-’ indicate the homozygous status and heterozygous status of the identified the DCAF17:c.1488_1489delAG respectively. **(B)** Sanger sequencing chromatograms of the DCAF17:c.1488_1489delAG in homozygous status (above) and heterozygous status (below).

**Figure 2:**
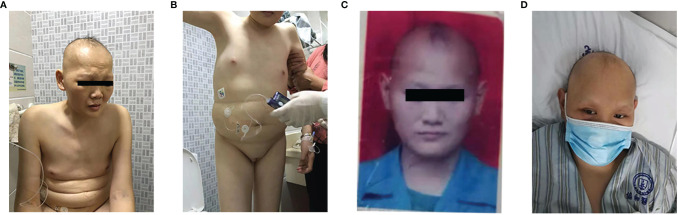
Photographs of the WSS patients. **(A, B)** The proband’s hair, eyebrows and eyelashes are sparse, and infantile external genitalia. **(C, D)** The brother of proband: childhood-onset hair thinning, His hair, eyebrows and eyelashes are further sparse in adulthood.

**Figure 3:**
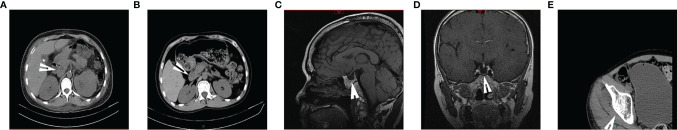
The images of the WSS patients. **(A)** Computed tomography of the abdomen of the proband demonstrated atrophy of the pancreas. **(B)** Computed tomography of the abdomen of the brother of proband showed uneven pancreatic density. **(C, D)** Pituitary MR of the brother of proband indicated the empty sella and none pituitary gland. **(E)** Hip CT of the brother of proband suggested osteoporosis that was not consistent with actual age. (arrows).

There were two affected individuals in this family line, whose parents were not consanguineously married (Family **Figure 1A**). The proband born on October 24, 1984, was found to have elevated blood glucose and edema throughout the body in November 2018 and was admitted to the hospital for treatment. Physical examination showed sparse scalp hair and eyebrows, absence of axillary and pubic hair, infantile external genitalia, generalized edema and intellectual disability (**Figures 2A, B**). Other data were normal, including bedside hearing and sensory testing (although formal assessment was not performed). Laboratory tests showed that the fasting glucose was 40.22 mmol/L (reference range: 3.9-6.1 mmol/L), and the glycosylated hemoglobin (HbA1c) was 13.8% (reference range: < 6.4%). Sex hormone tests revealed low levels of luteinizing hormone (LH), follicle stimulating hormone (FSH) and estradiol. Routine blood tests suggested that the patient had anemia and thrombocytopenia (**Table 1**). Insulin and C-peptide release tests showed that the patient’s insulin and C-peptide secretion had no peak (**Supplementary Table 1A**). The OGTT-based measures of insulin secretion index homeostasis model assessment- β (HOMA-β = 20 × Ins0/(Glu0-3.5) was low (**Table 1**). Thyroid hormone tests revealed normal thyroid-stimulating hormone (TSH), low free thyroxine (FT4), and a low free triiodothyronine level (FT3). Pancreatic CT indicated a slightly atrophied pancreas (**Figure 3A**). While ultrasound showed that the uterus was absent. The patient was treated with insulin injection, and the blood glucose was poorly controlled.

**Table 1:** Clinical features of affected individuals in the family.

Clinical features	Affected individuals		Normal reference range	
	II-1	II-2		
Sex	female	male		
Age(at first diagnosis of diabetes)	34	33		
Height (cm)	162	N/A		
Weight (kg)	54	45		
Clinical manifestations				
Alopecia	+	+		
Intellectual Disability	+	+		
Hypogonadism	+	+		
Diabetes Mellitus	+	+		
Anemia	+	+		
Thrombocytopenia	+	+		
Hypothyroidism	–	–		
Other Neurophysiology findings	–	–		
Sensorineural hearing loss	–	–		
Progressive extrapyramidal movements	–	–		
Laboratory tests				
Fasting blood glucose (mmol/L)	40.22	14.91	3.9-6.1	
HbA1c %	13.8	9.0	<6.4	
Islet beta-cell autoantibodies	N/A	–		
HOMA-β (%)	4.63	21.69		
IGF-1 (ng/ml)	N/A	43	115-320	
Hb (g/L)	81	105	115-150	
PLT (G/L)	63	89	125-350	
Sexual hormones			Male	Female (follicular phase)
Progesterone (ng/ml)	0.2	0.2		0.10-0.30
FSH (mIU/ml)	4.23	0.99	0.95-11.95	3.03-8.08
PRL (ng/ml)	11.17	5.4	3.46-19.40	5.18-26.53
Estradiol (pg/ml)	20	14	11-44	21-251
Testosterone (nmol/l)	1.6	0.89	4.94-32.01	0.38-1.97
LH (mIU/ml)	0.78	0.16	1.14-8.75	2.39-6.60
ECG abnormalities	+	+		

HbA1c, Glycated hemoglobin; Hb, Hemoglobin; PLT, Platelet; FSH, Follicle-stimulating hormone; PRL, Prolactin; LH, Luteinizing hormone; ECG, Electrocardiographic; N/A, not available; +, positive; -, negative.

### Case 2

The brother of the proband, who was born on October 29, 1986, was found to have elevated blood glucose in 2019 and admitted to our hospital for a closed fracture in January 2020. Physical examination also showed sparse scalp hair and eyebrows (**Figures 2C, D**), absence of axillary and pubic hair, hypotrophy testicles, small penis, and intellectual disability. Laboratory tests revealed that his fasting glucose was 14.91 mmol/L, HbA1c was 9.0%, he had negative insulin antibodies, low IGF-1 level of 43 ng/ml (reference range: 111–320 ng/ml), low testosterone and LH. Routine blood tests also suggested that the patient had anemia and thrombocytopenia. Insulin and C-peptide release tests showed low insulin, C-peptide secretion (**Supplementary Table 1B**) and low HOMA-β. Thyroid hormone tests revealed normal thyroid function. The patient exhibited an uneven pancreatic density by abdominal CT scan (**Figure 3B**), empty sella by pituitary MRI (**Figures 3C, D**), and osteoporosis that was not consistent with actual age by hip CT scan (**Figure 3E**). Before admission to the hospital, the patient had oral hypoglycemic agents to control glucose, and insulin pump treatment was given after admission to the hospital, but the glucose control was poor.

### Genetic Sequencing and Bioinformatics Analysis

5ml venous blood was collected from the proband (II-1, **Figure 1A**) and her family members (I-1, I-2, II-2 and II-3, **Figure 1A**). Whole Exome sequencing (WES) was performed on proband using the xGen^®^ Exome Research Panel v1.0 (IDT, USA) on the Illumina NovaSeq6000. Genomic DNA was extracted from whole blood, then sheared by sonication and hybridized for enrichment. And the library was enriched for target regions, sequencing was performed to generate 150 bp paired-end reads. To identify mutations, sequencing data was analyzed and annotated according to an in-house pipeline which we describe before (7). Based on the variant annotations, a series of prioritization strategies were applied to identify candidate variants associated with the phenotypes. The detailed steps were as follows: (1) excluding variants outside exonic and splicing regions; (2) excluding variants with minor allele frequency >=0.01 according to public databases (GnomAD, 1000 Genomes and ExAC); (3) excluding synonymous variants; (4) excluding variants not presenting damaging results in the function prediction from clinPred (8). To prioritize the most likely candidate disease-causing gene, all candidate genes were then ranked by Phenolyzer (9). Human Phenotype Ontology (HPO) Identifiers of the proband’s disease phenotype (anemia, HP:0001903; alopecia, HP:0001596; diabetes mellitus, HP:0000819; hypogonadism, HP:000013; intellectual disability, HP:0001249) were input as phenotype terms into Phenolyzer. To confirm the candidate disease-causing variant, we PCR-amplified the genomic DNA fragments in the proband (II-1) and her family members (I-1, I-2, II-4 and III-2), then sequenced them by Sanger sequencing. The PCR primer pairs were uses as followings: forward 5′–TCCTGGGTCCAGAGTTCTTT–3′, reverse 5′–TGGGGTGTTTGGCTAAAAGT–3′).

### Identification of Variants Associated With Disease Phenotypes of the Proband

Based on the aligned reads, 76,241 single nucleotide variants (SNVs) and 13,038 indels (deletions and insertions, <50bp) were identified by WES. After three filters of prioritization strategies, 271 variants from 244 genes were kept. Both these genes and HPO Identifiers of phenotypes of the proband were input Phenolyzer. Phenolyzer suggested that WSS was the most likely disease that can explain the syndrome phenotypes of the proband, and the DCAF17 gene was the candidate gene (**Supplementary Figure 1**). A homozygous deletion DCAF17:c.1488_1489delAG was detected at genomic position 172,337,546-172,337,547 of chromosome 2 (GRCh37/Hg19), resulting in frameshift in reading frame in exon 14 leading to premature stop codon (**Figure 1B**). The full-length DDB1 and CUL4 associated factor 17 contains 520 amino acids. The premature termination was predicted to cause a truncated protein comprising 506 amino acids. The first 495 residues corresponding to the normal protein and the other extra 11 derived from the frameshift deletion. Sanger sequencing analysis showed the identified c.1488_1489delAG (deletion of nucleotide AG in coding region 1488-1489) was segregated with the disease in this family, which was in homozygous status in the proband’s affected sibling (II-2) and heterozygous status in the proband’s unaffected parents (I-1 and I-2) and sibling (II-3) (**Figure 1A**). Additionally, the identified variant allele has been recorded in the database of gnomAD with an extremely low allele frequency at 3/251,124 (dbSNP rs778488574). According to the ACMG/AMP criteria, the raw homozygous frameshift variant DCAF17:c.1488_1489delAG was classified as a pathogenic variant for WSS.

## Discussion

The clinical features of WSS patients are not entirely consistent. Most patients present extrapyramidal symptoms, intellectual disability, hypogonadism, alopecia and diabetes. We found two patients in a Chinese national lineage who exhibited diabetes mellitus, intellectual disability, hair thinning, and hypogonadism but no symptoms of progressive extrapyramidal dysfunction. The boosted whole exome gene sequencing technique suggested the presence of the NM_025000.4:c.1488_1489delAG variant in the DCAF17 gene (sequencing **Figure 1B**), which established the diagnosis of WSS in this lineage. We further performed insulin and C-peptide release tests and found pancreatic β cell functional impairment in both patients. We identified a WSS lineage in China with a novel DCAF17 gene mutation pattern and suggested that pancreatic β cell functional impairment might be one of the major mechanisms in the pathogenesis of WSS diabetes.

The prevalence of diabetes mellitus in patients with WSS has been reported to be as high as 66%, and it mostly starts from adolescents to young adults (2). There are few studies on the pathogenesis of diabetes mellitus in WSS. Sendur SN et al. (2019) analyzed adolescent-onset diabetes mellitus in WSS case and found no significant increase in C-peptide release levels (10). Rachmiel M et al. (2011) detected low C-peptide levels and pancreatic atrophy when imaging some patients with abnormally elevated blood glucose (11). Both patients with WSS that we reported had diabetes in young adulthood with reduced insulin and C-peptide levels, negative insulin-related antibodies, and pancreatic atrophy from abdominal CT. HOMA-β was applied to assess β-cell function, HOMA-β was less than 100% and lower than type 2 diabetes in the two cases (12). Low insulin, low C-peptide levels, low HOMA-β and an atrophic pancreas suggest that WSS patients may have pancreatic β cell function defects. Therefore, we speculate that the DCAF17 gene is important for the maintenance of pancreatic β cell function. DCAF17 gene mutations may cause β cell functional defects, which may be one of the main mechanisms in the pathogenesis of diabetes in patients with WSS. However, the underlying pathological mechanism requires further study.

Thirty percent of individuals, typically around age 20 years, have hypothyroidism in patients with WSS (2). The proband showed low FT3 and FT4 with normal TSH. We observed decreased cortisol secretion in patients, which might indicate hypopituitarism.

In these two patients, we reported that the homozygous nucleotide variant changed the codon for the compilation of amino acid Arg 429 to Ser (NP_079276.2:p.Arg496SerfsX12), which prematurely terminated the peptide chain synthesis as a deletion variant and resulted in a truncated nonfunctional protein. These two patients had no significant extrapyramidal symptoms, dystonia or hearing loss. However, the proband’s brother had osteoporosis not previously described in this WSS. Their clinical features are not entirely consistent with previously reported cases. The length of the truncated proteins does not parallel the phenotypes reported in patients with WSS. Ali RH et al. (2016) proposed that the cellular clearance of the truncated DCAF17 version rescued the unexpected outcomes of cellular events through nonsense-mediated decay (NMD) of mRNAs, which could be perturbed by an inefficient interaction of the truncated protein (13). A truncated DCAF17 protein with missing domains and motifs that are necessary to interact with the DDB1-CUL4 ubiquitin ligase complex and ultimately recruit substrates was formed.

The treatment of WSS patients is currently individualized and managed in a multi-disciplinary pattern (2). Since specific mechanisms and drug targets that are responsible for the pathogenesis of WSS remain unclear, the current therapeutic principles are only symptomatic treatments. For hypogonadism, estrogen or androgen replacement therapy is indicated. For patients with WSS complicated by diabetes, the main treatment principle is to control the blood glucose levels and delay the development of diabetes-related complications. We have found that β cell function defects may be the main mechanism for the development of diabetes in WSS, which suggests that insulin should probably be the main treatment for patients with WSS when diabetes occurs.

The current study has certain limitations. First, the number of cases in this study slightly less, to reflect the results of the accuracy and comprehensiveness. Second, there was no dynamic detection of changes in hormone levels. Third, in this study the indices of insulin sensitivity and secretion were obtained from OGTT, not the euglycemic-hyperinsulinemic and the hyperglycemic clamp technique. Fourth, further experimental studies are necessary to reveal the exact pathogenic mechanisms of how these genes affect different tissues.

## Conclusions

Woodhouse-Sakati syndrome is a very rare autosomal recessive hereditary metabolic disorder. We observed the presence of impaired pancreatic (β cells) and gonadal function in patients with WSS. Further tests revealed the presence of low insulin secretion, which suggests that β cell functional defects due to DCAF17 gene mutation are among possible mechanisms for the development of diabetes in WSS. In this study, we reported a new pathogenic sequence variant in DCAF17, NM_025000.4:c.1488_1489delAG, which may expand the spectrum of DCAF17 variants. In addition to the endocrine findings of hypogonadism and diabetes mellitus, the decreased β cell function and osteoporosis observed are novel findings with unknown mechanisms. We have no neurologic findings of progressive extrapyramidal movements, or moderate bilateral postlingual sensorineural hearing loss other than intellectual disability. Whether phenotypes are associated with different mutation types requires further study.

## Data Availability Statement

The datasets presented in this study can be found in online repositories. The names of the repository/repositories and accession number(s) can be found in the article/**Supplementary Material**.

## Ethics Statement

This study was approved by the ethics committee of Tongji Medical College, Huazhong University of Science and Technology, Wuhan, P.R China and informed consent was obtained from the patients and their parents. Written informed consent was obtained from the individual(s) for the publication of any potentially identifiable images or data included in this article.

## Author Contributions

All the authors have contributed significantly. HL designed the study. MZ, NS, and JZ wrote the manuscript. MZ, NS, JZ, YC, SW, KX, ZC, KQ, and FZ collected and analyzed the clinical data, participated in discussion. HL supervised the study and corrected the manuscript. All authors contributed to the article and approved the submitted version.

## Funding

This work was supported by the grant from the National Natural Science Foundation of China (grant numbers 81974111 to HL).

## Conflict of Interest

The authors declare that the research was conducted in the absence of any commercial or financial relationships that could be construed as a potential conflict of interest.

## Publisher’s Note

All claims expressed in this article are solely those of the authors and do not necessarily represent those of their affiliated organizations, or those of the publisher, the editors and the reviewers. Any product that may be evaluated in this article, or claim that may be made by its manufacturer, is not guaranteed or endorsed by the publisher.

## Supplementary Material

Supplementary Figure 1 A snapshot of the output from the Phenolyzer. **(A)** The interactive gene-disease-term network indicates the WSS is the most likely disease that can explain the syndrome phenotypes, which including anemia, alopecia, diabetes mellitus, hypogonadism, and intellectual disability. **(B)** The interactive gene-disease-term network indicates the DCAF17 is the candidate gene.
