# Corrigendum: Case Report: A Chinese Family of Woodhouse-Sakati Syndrome With Diabetes Mellitus, With a Novel Biallelic Deletion Mutation of the DCAF17 Gene

**DOI:** 10.3389/fendo.2022.856002

**Published:** 2022-02-21

**Authors:** Min Zhou, Ningjie Shi, Juan Zheng, Yang Chen, Siqi Wang, Kangli Xiao, Zhenhai Cui, Kangli Qiu, Feng Zhu, Huiqing Li

**Affiliations:** ^1^ Department of Pulmonary and Critical Care Medicine, Tongji Hospital, Tongji Medical College, Huazhong University of Science and Technology, Wuhan, China; ^2^ Key Laboratory of Respiratory Diseases, National Ministry of Health of the People’s Republic of China and National Clinical Research Center for Respiratory Disease, Wuhan, China; ^3^ Department of Endocrinology, Union Hospital, Tongji Medical College, Huazhong University of Science and Technology, Wuhan, China; ^4^ Hubei Provincial Clinical Research Center for Diabetes and Metabolic Disorders, Wuhan, China; ^5^ Clinic Center of Human Gene Research, Union Hospital, Tongji Medical College, Huazhong University of Science and Technology, Wuhan, China; ^6^ Department of Cardiology, Union Hospital, Tongji Medical College, Huazhong University of Science and Technology, Wuhan, China

**Keywords:** Woodhouse–Sakati syndrome, diabetes, intellectual disability, alopecia, hypogonadism

In the article as published originally, there was a typographical error in the caption for **Figure 3B**.

**Figure 3:**
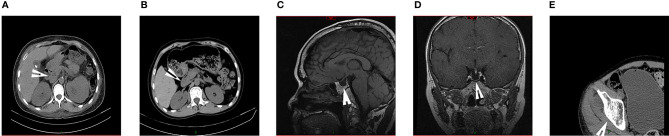
The images of the WSS patients. **(A)** Computed tomography of the abdomen of the proband demonstrated atrophy of the pancreas. **(B)** Computed tomography of the abdomen of the brother of proband showed uneven pancreatic density. **(C, D)** Pituitary MR of the brother of proband indicated the empty sella and none pituitary gland. **(E)** Hip CT of the brother of proband suggested osteoporosis that was not consistent with actual age. (arrows).

The sentence “Computed tomography of the, bdomen of the brother of proband showed uneven pancreatic density” should be “Computed tomography of the abdomen of the brother of proband showed uneven pancreatic density”.

The authors apologize for this error and state that this does not change the scientific conclusions of the article in any way. The original article has been updated.

In the article as published originally, there was a typographical error in **Table 1**. The normal reference range for IGF-1 was incorrectly presented as “115-323 ng/ml”; the correct adult reference range is “115-320 ng/ml”.

**Table 1:** Clinical features of affected individuals in the family.

	Affected individuals		Normal reference range	
Clinical features	II-1	II-2		
Sex	female	male		
Age(at first diagnosis of diabetes)	34	33		
Height (cm)	162	N/A		
Weight (kg)	54	45		
Clinical manifestations				
Alopecia	+	+		
Intellectual Disability	+	+		
Hypogonadism	+	+		
Diabetes Mellitus	+	+		
Anemia	+	+		
Thrombocytopenia	+	+		
Hypothyroidism	–	–		
Other Neurophysiology findings	–	–		
Sensorineural hearing loss	–	–		
Progressive extrapyramidal movements	–	–		
Laboratory tests				
Fasting blood glucose (mmol/L)	40.22	14.91	3.89-6.4	
HbA1c %	13.8	9.0	<6.4	
Islet beta-cell autoantibodies	N/A	–		
HOMA-β (%)	4.63	21.69		
IGF-1 (ng/ml)	N/A	43	115-320	
Hb (g/L)	81	105	115-150	
PLT (G/L)	63	89	125-250	
Sexual hormones			Male	Female (follicular phase)
Progesterone (ng/ml)	0.2	0.2		0.10-0.30
FSH (mIU/ml)	4.23	0.99	0.95-11.95	3.03-8.08
PRL (ng/ml)	11.17	5.4	3.46-19.40	5.18-26.53
Estradiol (pg/ml)	20	14	11-44	21-251
Testosterone (nmol/l)	1.6	0.89	4.94-32.01	0.38-1.97
LH (mIU/ml)	0.78	0.16	1.14-8.75	2.39-6.60
ECG abnormalities	+	+		

HbA1c, Glycated hemoglobin; Hb, Hemoglobin; PLT, Platelet; FSH, Follicle-stimulating hormone; PRL, Prolactin; LH, Luteinizing hormone; ECG, Electrocardiographic; N/A, not available; +, positive; -, negative.

The authors apologize for this error and state that this does not change the scientific conclusions of the article in any way. The original article has been updated.

In the article as published originally, there was a typographical error in the section *Case Description*, subsection *Case 2*. The IGF-1 range for Case 2 was incorrectly presented as “111-549 ng/ml”; the correct adult reference range is “115-320 ng/ml”.

The authors apologize for this error and state that this does not change the scientific conclusions of the article in any way. The original article has been updated.

## Publisher’s Note

All claims expressed in this article are solely those of the authors and do not necessarily represent those of their affiliated organizations, or those of the publisher, the editors and the reviewers. Any product that may be evaluated in this article, or claim that may be made by its manufacturer, is not guaranteed or endorsed by the publisher.

