# Supplementary figures and images for: Case Report: A Chinese Family of Woodhouse-Sakati Syndrome With Diabetes Mellitus, With a Novel Biallelic Deletion Mutation of the DCAF17 Gene
Source: Front Endocrinol (Lausanne). 2021 Dec 23;12:770871. doi: 10.3389/fendo.2021.770871 (PMC8734028; doi:10.3389/fendo.2021.770871)

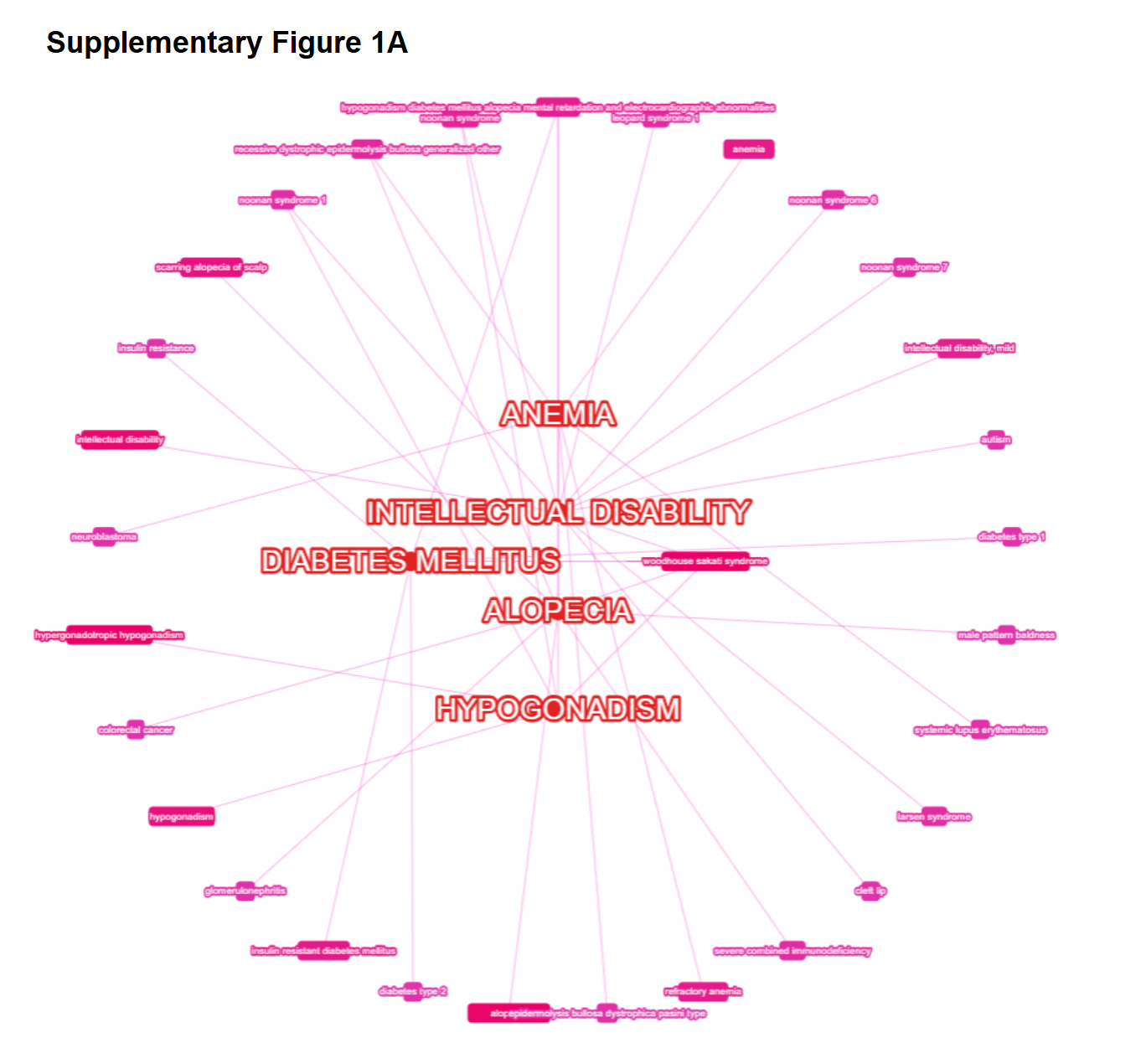

Supplement: Supplementary Figure 1 — A snapshot of the output from the Phenolyzer. (A) The interactive gene-disease-term network indicates the WSS is the most likely disease that can explain the syndrome phenotypes, which including anemia, alopecia, diabetes mellitus, hypogonadism, and intellectual disability. (B) The interactive gene-disease-term network indicates the DCAF17 is the candidate gene. [file Image_1.png]

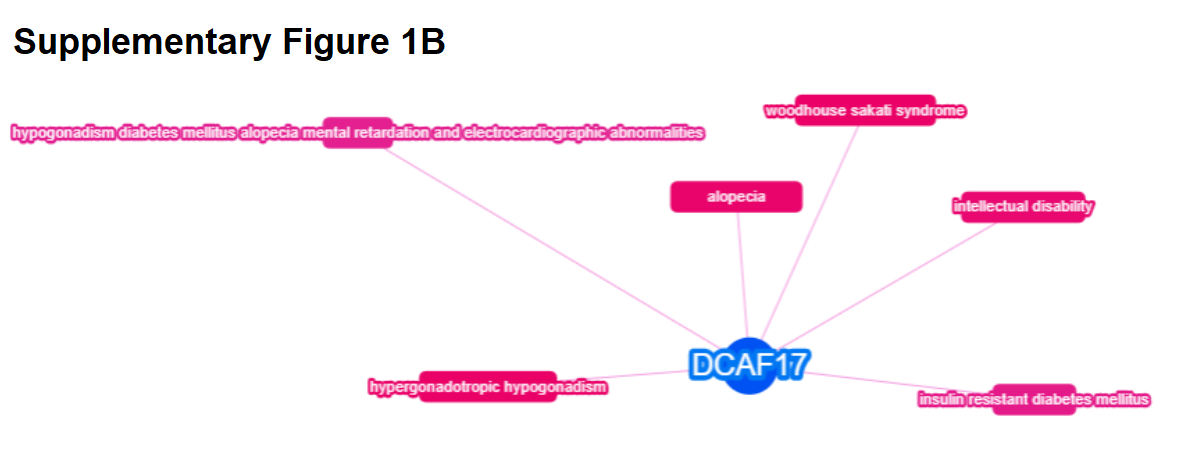

Supplement: Supplementary file 2 [file Image_2.png]
