# Supplementary material for: Case Report: A Chinese Family of Woodhouse-Sakati Syndrome With Diabetes Mellitus, With a Novel Biallelic Deletion Mutation of the DCAF17 Gene
Source: Front Endocrinol (Lausanne). 2021 Dec 23;12:770871. doi: 10.3389/fendo.2021.770871 (PMC8734028; doi:10.3389/fendo.2021.770871)
Supplement: Supplementary file 3 [file Table_1.docx]

Supplementary Table 1 The β cell function test results of two affected individuals. Both WSS patients took 75g glucose orally. The venous blood was drawn before drinking sugar water and 30min, 60min, 120min, 180min after the first mouthful of sugar water. The blood glucose, insulin and C peptide level at these points were tested. Abbreviations: IRT, Insulin releasing test; CRT, C peptide release test.

1. The proband：

| Blood test time (min) | Blood glucose (mmol/L) | Insulin (μIU/mL) | C-Peptide (ng/ml) |
| --- | --- | --- | --- |
| 0 | 18.19 (reference range: 3.9-6.1) | 3.4 (reference range: 1.8-11.8) | 1.31 (reference range: 0.78-5.19) |
| 30 | 19.1 | 3.9 | 1.33 |
| 60 | 21.6 | 5.0 | 1.53 |
| 120 | 22.9 | 4.3 | 1.49 |
| 180 | 26.9 | 3.5 | 1.42 |

1. The brother of the proband：

| Blood test time (min) | Blood glucose (mmol/L) | Insulin (μIU/mL) | C-Peptide (ng/ml) |
| --- | --- | --- | --- |
| 0 | 10.6 (reference range: 3.9-6.1) | 7.7 (reference range: 1.8-11.8) | 1.23 (reference range: 0.78-5.19) |
| 30 | 11.84 | 6.4 | 1.09 |
| 60 | 16.41 | 9.5 | 1.37 |
| 120 | 20.77 | 16.3 | 2.33 |
| 180 | 19.32 | 19.3 | 3.07 |
